# Available Wireless Sensor Network and Internet of Things testbed facilities: dataset

**DOI:** 10.12688/openreseurope.15176.2

**Published:** 2023-11-28

**Authors:** Janis Judvaitis, Valters Abolins, Amr Elkenawy, Kaspars Ozols

**Affiliations:** 1 Institute of Electronics and Computer Science, Riga, Latvia

**Keywords:** Testbed facility; Data set; Wireless Sensor Networks; WSN; Internet of Things; IoT

## Abstract

The availability of data is an important aspect of any research as it determines the likelihood of the study’s commencement, completion, and success. The Internet of Things and Wireless Sensor Networks technologies have been attracting a huge amount of researchers for more than two decades, without having a consolidated or unified source that identifies and describes available Internet of Things and Wireless Sensor Network testbed facilities. In this paper, a dataset including 41 distinct testbed facilities is described. These testbed facilities are classified according to their key features such as Device Under Test (DUT) type, mobility, access level, facility count, connection/interaction interfaces, and other criteria. The systematic review process resulting in the gathered data set consisted of three filtering phases applied to relevant articles published between the years 2011 and 2021 as obtained from the Web of Science and SCOPUS databases.

## Plain language summary

In this Data Note, we present data collected for the purpose of carrying out a systematic review of the available Wireless Sensor Network and Internet of Things testbed facilities. The data was collected through multiple stages and in each stage, the pre-defined criteria were applied. We provide a dataset describing the hardware and software aspects of Wireless Sensor Networks and Internet of Things testbed facilities available in the market and scientific community. The data were gathered through an extensive systematic review process of scientific articles published between the years 2011 and 2020. The review aims to obtain good-quality data for people who are actively researching the Internet of Things or Wireless sensor Network testbed facilities or anyone who is interested in that field.

## Introduction

Nowadays, the Internet of Things (IoT) term is often used due to its significant role in enabling smart interactions between machines, sensors, and the environment ^1^. The IoT approach aims to orchestrate a set of "things" or technologies such as sensors, actuators, radio frequency identification (RFID) tags, near field communications (NFC), and machine-to-machine (M2M) communications, by means of network protocols, in order to achieve the required goal by the developed IoT system ^1, 2^. IoT applications and use cases can vary from manufacturing and agriculture to healthcare and transportation, with a wide spectrum in between ^1^. One specific example of an IoT application could be an indoor localization system using an IoT testbed as described by Elkenawy *et al.* ^3^.

To build a complete IoT system as a Wireless Sensor Network (WSN) infrastructure, an efficient prototyping procedure must be carried out as a first step. Testbed facilities are a great tool for prototyping purposes compared to other simulation or emulation tools, as they represent the real-world conditions more precisely, which in turn speeds up the development process and could make the process of developing WSNs, making debugging and testing less time consuming ^4, 5^. A plain definition for the WSN testbed facility would be a realistic/physical environment consisting of a large number of permanently deployed sensor nodes (25+ nodes according to Ruskuls *et al.* ^6^) with a software backend that provides a basic set of functionalities such as node reprogramming and remote interaction. On top of that, a lot of testbed facilities provide additional features such as data logging, experiment scheduling ^7^, energy metering ^8^, *etc.*

A good dataset demonstrating testbed facilities, in terms of facilities’ capabilities versus market needs, would provide guidelines with regard to design choices for testbed facilities and provide useful information during the creation and execution of scientific experiments, in addition to fueling innovative solutions and remarkable competition ^9^ within the market. To the best of our knowledge, a comprehensive and up-to-date dataset for testbed facilities is a scarce resource that is not yet available to the research community. An illustrative example of this is that querying "IoT testbed" in Google Dataset Search would result in:

- Data generated during a testbed experiment;
- Specifications of a testbed facility;
- Description of a cluster of testbed facilities (e.g. cybersecurity testbeds category).

A broader survey has been done by Judvaitis *et al.* ^10^ by extracting information about 3059 sensor network deployments according to different categories, which is one of the few existing attempts for synthesizing a complete dataset for actual sensor network deployments. This data set was gathered with the aim of providing a definite overview of existing Wireless Sensor Networks and Internet of Things testbed facilities available for scientific and industrial use and identifying possible gaps to be filled by future testbed facility developments.

The research question addressed by this article is how many and what testbed facilities are available and how do they compare against one another.

## Methods

This dataset was gathered following the Preferred Reporting Items for Systematic Review and Meta-Analysis (PRISMA) ^11^ checklist. The initial search was done in two databases *Scopus* and *Web of Science* (WoS) using the following queries:

Scopus: TITLE ( testbed ) AND TITLE-ABS-KEY ( wsn OR iot OR "sensor network*" OR "internet of thing*" ) AND SUBJAREA ( comp ) AND PUBYEAR < 2021 AND PUBYEAR > 2010

WoS: TI = ("testbed") AND (AB = (wsn OR iot OR "sensor network*" OR "internet of thing*") or AK= (wsn OR iot OR "sensor network*" OR "internet of thing*") ) and SU="Computer Science" and py =(2011–2020)

The raw results returned 346 articles from Scopus and 176 articles from WoS. After 163 duplicates were removed, 359 unique articles were left for further analysis. In the phases described below each article was mainly processed by one reviewer independently. For each reviewer in each phase articles were assigned randomly. This minimized the risk of biases while assigning the articles to a particular reviewer. The confusing and difficult-to-evaluate articles were discussed in weekly meetings or re-evaluated by another reviewer.

### First phase

In this phase, only the abstracts of all 359 were analyzed. The aim was to filter articles that did not contain a description of the testbed facility. With a testbed facility, we understand the following: the facility provides remote access to the embedded hardware which can be used freely without any restrictions regarding the usability or functionality, and it should meet the following minimal requirements:

- Designed to run a variety of different experiments, where the devices under test are completely controlled by the user;
- Users do not need physical access to the hardware- reprogramming or any other interaction with software can be performed remotely;
- Provide a user interface specifically designed for testbed facility purposes.

Access to the testbed facility can be restricted and is not necessary for a testbed facility to qualify, so it does not necessarily need to be public. Articles containing descriptions of improvements to testbed facilities were also to be included, even if they did not contain a description of the testbed facility itself. The guidelines for researchers who did the screening were the following:

(a) Open the article you are going to evaluate.
(b) Read the abstract.
(c) Make a decision about the article, does it contain a testbed facility description, and, if so, mark it as included for further analysis.
(d) Mark the article as screened.
(e) Go to the next article.

We used Mendeley ( https://www.mendeley.com/) to quickly share the progress between the team members and automatically obtain the abstracts for the articles. 9 team members (3 of which are the authors) screened the abstracts. As a result of the first phase, 170 articles were dropped, as they did not contain a testbed facility description. A total of 189 articles were left for further analysis.

### Second phase

The aim of this phase was to filter out non-testbed facility articles and group the articles by distinct testbed facilities, assuming that there can be multiple articles per testbed facility. In this phase, the full article was screened, and, using the same criteria as in the previous phase, the article was marked as “containing testbed facility” or not. We used a Microsoft Excel worksheet to track the progress and split the work between researchers. 4 team members (3 of which are the authors) screened full texts of articles. After the second phase, 125 articles were marked as not containing a testbed facility description.

### Third phase

In this phase, to minimize the risk of missed articles in the second phase, all 189 articles (the same articles as in the previous phase) were processed and predefined values (with their descriptions) were extracted: device under test (DUT), Sensors (the type), location (generic description of the place where the DUTs are located), mobility (can the DUTs physically move while the experiment is ongoing), architecture (the internal design of the testbed facility), workstations (workstation is a DUT-to-server relay, typically a Linux-capable device that forwards the data and commands between server and DUTs), DUT location accuracy (what is the precision to which the user knows the DUT location), cost of implementation (if provided), deployment options (is the testbed suited and intended to deploy outside of the laboratory), facility count (assuming there might be more than one facility), functionality (the features this testbed facility provides to users), access level (what is the level of user control), user interface (the type of used UI), assistive tools (any tools designed to facilitate or improve the user experience), DUT connection and interaction interfaces (physical connections available), and whether the testbed facility is available as Open Source.

**Figure 1.**
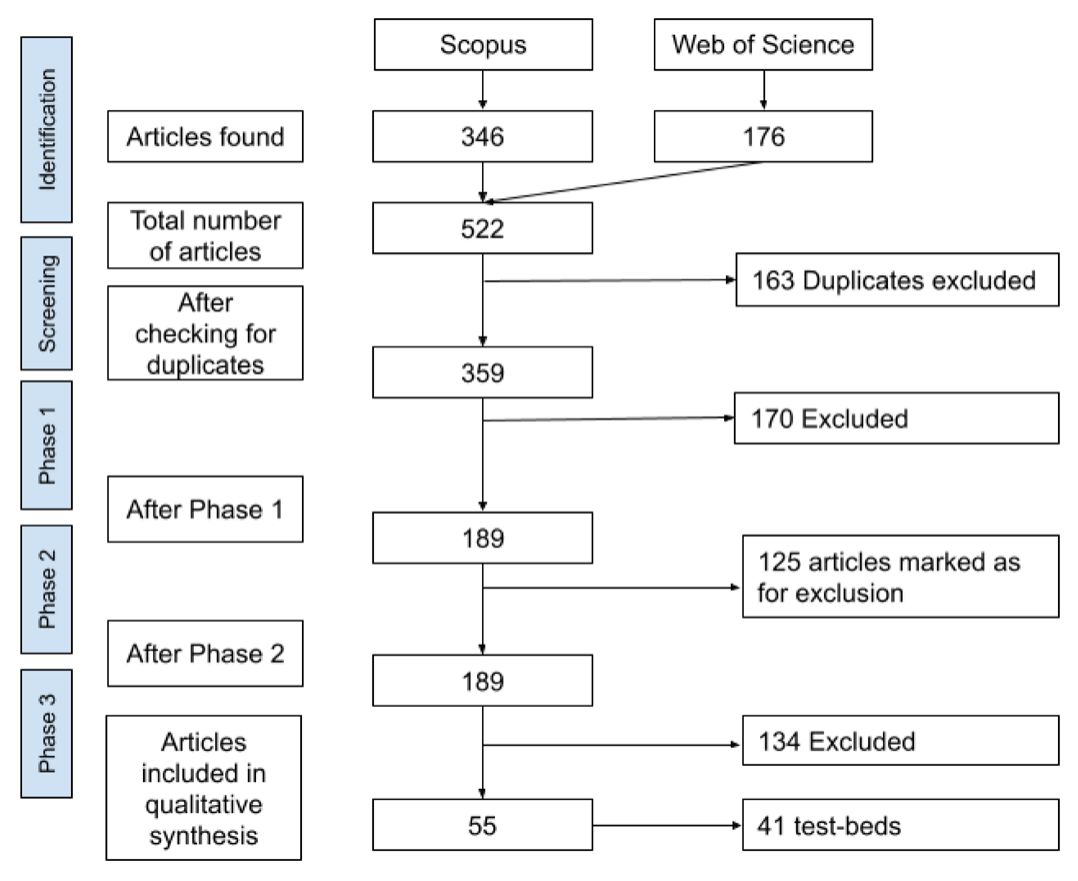
Flow diagram overview of the systematic review methodology and results at each phase.

All articles were processed again by the whole team to minimize the risk of error in the previous phase. We used JSONForms ( https://jsonforms.io/) to make the extraction process smoother and obtain machine-readable data in JSON format. After the third phase, 134 articles (125 from the previous phase + an extra 9 articles) were excluded and 55 articles were included in the dataset. There were multiple testbeds with more than one article describing them. In total, 41 unique testbed facilities were described in 55 articles. An overview of all the systematic review phases described above is summarized in a flow diagram in Figure 1, depicting the whole process.

## Data processing

As the data describing testbed facilities contains data entries that are not machine-readable, the nature of the resulting data set does not allow processing using programming tools such as Python/Jupyter Notebooks, it can only be viewed as a textual compilation of different specifics about the evaluated testbed facilities. As a remedy to this situation, we compiled a subset of all the extracted data unifying and simplifying the extracted values to be more easy to use. The unifying process and actions taken were discussed with the team members to gain a unified vision of the process. Still, the process itself was done by the leading researcher to minimize the interpretation difference risks. After this process, the team members reviewed the unified dataset. The newly obtained machine-readable data set is in unified JSON format and thus can be read and analyzed by any modern programming language script or data analytics application, the data set includes some initial analytic scripts written in Python. The machine-readable data set contains less information overall, as similar values were merged for increased readability. In the published dataset we have included both versions, together with the tools used to obtain the datasets.

## Ethical approval and consent

Ethical approval and consent were not required.

## Data Availability

Zenodo: *Available Wireless Sensor Network and Internet of Things testbed facilities: dataset,* https://doi.org/10.5281/zenodo.7157221 ^12^. This project contains the following underlying data: **raw_dataset** contains the files for each article containing a testbed facility description with the extracted information in json format; **json** contains json files for each testbed facility processed with the aim of improving the machine readability of the dataset; **output.json** contains a json array with the names of extracted features and the list of testbed facility IDs corresponding to the json file names with such features; **feature_extraction.ipynb** contains the script used for the initial analysis of the JSON files about testbed facilities. Zenodo: *Available Wireless Sensor Network and Internet of Things testbed facilities: dataset,* https://doi.org/10.5281/zenodo.7157221 ^12^. This project contains the following extended data: **Supplementing_info** */* **raw_exports** */*contains the raw list of articles exported from SCOPUS and WoS databases; **Supplementing_info** */* **processed** */* **1-combined.xlsx** contains the combined list of articles; **Supplementing_info** */* **processed** */* **2-deduplicated.xlsx** contains the deduplicated list of articles; **Supplementing_info** */* **jsonforms** */*contains the web-based tool used for testbed facility feature extraction in the Third phase; For further details on the structure and contents of each folder please view the README.md file. The dataset is licensed under CC-BY 4.0 International (CC-BY 4.0) Public Domain Dedication license.
