# Supplementary material for: Available Wireless Sensor Network and Internet of Things testbed facilities: dataset
Source: Open Res Eur. 2023 Nov 28;2:127. Originally published 2022 Nov 24. [Version 2] doi: 10.12688/openreseurope.15176.2 (PMC10794863; doi:10.12688/openreseurope.15176.2)
Supplement: Peer review history

# Reviewer report: Reviewer response for version 2

**DOI:** 10.21956/openreseurope.18263.r36687

**Published:** 2024-02-26

**Authors:** Mohamed-Lamine Messai

**Affiliations:** 1 University of Lyon 2, Lyon, France

In this submission, published scientific papers on sensor and IoT networks testbeds are used to construct a dataset. However, the aim of constructing such a dataset is not clear. Additionally, the use of IoT context depends on the application requirements. Does studying the availability of testbed facilities necessitate a dataset?

 A clear definition of an IoT system is needed; is an IoT system a WSN connected to the Internet? In my opinion, and for more clarification for the readers, the reference section should include the 41 references to testbeds.

 The design of sensor and IoT networks is application-sensitive, a point the paper does not consider. For example, in some kinds of applications, mobility is used, but not all testbeds offer this possibility.

 The authors present three minimal requirements needed to use a testbed, but in my opinion, other requirements need to be added.

 For the 170 articles that were dropped, have the authors checked that the descriptions are not available on testbed websites, GitHub, etc.?

 Why use the same criterion "containing testbed facility" for both phases 1 and 2? These two phases could be merged.

 The transition from Phase 3 to Phase 4 requires more details.

 The authors do not indicate why their dataset is useful. Why is the URL of the testbeds' websites not a feature in the dataset (FIT IoT-Lab, etc.)?

 Finally, the paper lacks a conclusion.

Are sufficient details of methods and materials provided to allow replication by others?

Partly

Is the rationale for creating the dataset(s) clearly described?

Partly

Are the datasets clearly presented in a useable and accessible format?

Partly

Are the protocols appropriate and is the work technically sound?

Partly

Reviewer Expertise:

Wireless sensor and IoT Networks, ML & Cybersecurity, datasets

I confirm that I have read this submission and believe that I have an appropriate level of expertise to state that I do not consider it to be of an acceptable scientific standard, for reasons outlined above.

# Reviewer report: Reviewer response for version 2

**DOI:** 10.21956/openreseurope.18263.r36699

**Published:** 2024-01-17

**Authors:** Ousmane Thiare

**Affiliations:** 1 Gaston Berger University, Saint-Loui, Senegal

This article makes a great contribution to the Internet of Things and wireless sensor networks, particularly in the availability of testbeds with a data set.

 The question of the availability of complete data on which researchers can work has always been a crucial issue in research related to IoT and wireless sensor networks. It's worth noting in this paper, however, how a rigorous selection has been made to really focus on available resources from reliable and different sources. This is an assurance and also brings precision and reliability for the benefit of the research community in this field.

 The paper does address the collection and classification of data, but doesn't really say how this data should be exploited for future research.

 Personally, this article will enable the research community to use testbeds for the transition from simulations to real deployments. This will prevent researchers from using more simulation tools rather than real deployments.

Are sufficient details of methods and materials provided to allow replication by others?

Partly

Is the rationale for creating the dataset(s) clearly described?

Yes

Are the datasets clearly presented in a useable and accessible format?

Yes

Are the protocols appropriate and is the work technically sound?

Yes

Reviewer Expertise:

Internet of things, Wireless sensor networks, distributed systems

I confirm that I have read this submission and believe that I have an appropriate level of expertise to confirm that it is of an acceptable scientific standard.

# Reviewer report: Reviewer response for version 2

**DOI:** 10.21956/openreseurope.18263.r36476

**Published:** 2024-01-05

**Authors:** Ihsan Ali

**Affiliations:** 1 University of Malaya, Kuala Lumpur, Wilayah Persekutuan, Malaysia

Most of my comments are addressed by the authors. The paper may be accepted for indexing.

Are sufficient details of methods and materials provided to allow replication by others?

Partly

Is the rationale for creating the dataset(s) clearly described?

Yes

Are the datasets clearly presented in a useable and accessible format?

Partly

Are the protocols appropriate and is the work technically sound?

Partly

Reviewer Expertise:

NA

I confirm that I have read this submission and believe that I have an appropriate level of expertise to confirm that it is of an acceptable scientific standard.

# Reviewer report: Reviewer response for version 1

**DOI:** 10.21956/openreseurope.16415.r33751

**Published:** 2023-09-15

**Authors:** Ihsan Ali

**Affiliations:** 1 University of Malaya, Kuala Lumpur, Wilayah Persekutuan, Malaysia

The article presents a valuable contribution to the field of Internet of Things (IoT) and Wireless Sensor Networks (WSN) research by addressing the crucial issue of testbed facility availability. Here is a critical review of the article:

 On the positive aspects of the article, the study underscores the importance of testbed facilities in IoT and WSN research. Availability of comprehensive data on these facilities is essential for researchers to plan and conduct experiments effectively. Moreover, the systematic approach to collecting and classifying testbed facilities based on key features such as DUT type, mobility, access level, and connection interfaces is commendable. This classification provides a clear and organized overview of the available resources. Furthermore, the article transparently outlines the systematic review process used to gather the dataset. The use of well-established databases like Web of Science and SCOPUS adds credibility to the data collection process. Finally, the willingness to share the collected dataset is a positive aspect of the article. Making this data available to the research community can significantly benefit scholars and practitioners in the IoT and WSN domains.

 My critical analysis and few reservations are as follows.

 While the article broadly addresses the availability of testbed facilities, it could benefit from a more explicit focus on specific research questions or objectives related to these facilities. Defining the research goals and context more clearly would enhance the article's relevance.

 More experimentation is needed on the collection of data. Synthetic data always raises several concerns. The article briefly mentions the dataset but lacks an actual presentation of the dataset, making it difficult for readers to access and utilize this valuable resource. Providing a link or access to the dataset itself would greatly enhance the article's usefulness.

 On the contrary, the article does not discuss any limitations or potential biases in the dataset or the systematic review process. Acknowledging these limitations would provide a more balanced perspective for researchers using this data.

 The article mainly focuses on data collection and classification, it could expand on potential future research directions or applications of the gathered dataset. This would help guide researchers interested in using the data for various purposes.

 Finally, the article lacks a concise conclusion section summarizing the key findings, contributions, and the broader implications of the dataset for the IoT and WSN research community. A well-structured conclusion would enhance the article's overall impact.

 In summary, the article addresses a significant gap in IoT and WSN research by compiling a dataset of testbed facilities. Its systematic approach and transparency in data collection are commendable. However, to maximize its value, the article should clarify its research objectives, present the dataset itself, acknowledge limitations, suggest future research directions, and provide a robust conclusion. These improvements would make the article an even more valuable resource for the academic community.

Are sufficient details of methods and materials provided to allow replication by others?

Partly

Is the rationale for creating the dataset(s) clearly described?

Yes

Are the datasets clearly presented in a useable and accessible format?

Partly

Are the protocols appropriate and is the work technically sound?

Partly

Reviewer Expertise:

WSNs, IoT, Sensor Cloud, Cloud Computing

I confirm that I have read this submission and believe that I have an appropriate level of expertise to confirm that it is of an acceptable scientific standard, however I have significant reservations, as outlined above.

# Response

**Published:** 2023-11-24

**Authors:** Valters Abolins

**Affiliations:** Institute of Electronics and Computer Science, Latvia

Dear Ihsan Ali, thank you for the provided review, comments and suggestions, here is our answers to them:   We have added a research objective description in the introduction section of the article. The dataset itself is available and is mentioned in the article in the section "Data availability" as bibliography reference [12]. It is published in the well recognized Zenodo platform and can be found here: https://doi.org/10.5281/zenodo.7157221 We are not aware of any limitations or biases in the dataset or the systematic review process, if we know them, we would have addressed them. With the only exception being the queries executed on the SCOPUS and Web of Science databases and the limits they impose on the found results, but they are published in the article. As for the potential future research directions, by the time of writing it was only a plan, but as of now, the complete systematic review about the published dataset is completed and published by us. The article by written by us "Testbed Facilities for IoT and Wireless Sensor Networks: A Systematic Review" can be found here: https://doi.org/10.3390/jsan12030048 Just for the clarification, this dataset publication is only about the gathering and compiling of the dataset and the systematic review I mentioned deals with the analysis of the gathered data and trends.

# Reviewer report: Reviewer response for version 1

**DOI:** 10.21956/openreseurope.16415.r33754

**Published:** 2023-07-28

**Authors:** Prasanth A

**Affiliations:** 1 Venkateswara College of Engineering, Sriperumbudur, Tamil Nadu, India

This article contains new and significant information adequate to justify the publication. However, the reviewer has some suggestions, as listed below:

1. Some sentences have grammatical errors. So, the authors need to check the grammatical errors throughout the paper.
2. Figure 1 should be properly explained in the main text.

Are sufficient details of methods and materials provided to allow replication by others?

Partly

Is the rationale for creating the dataset(s) clearly described?

Yes

Are the datasets clearly presented in a useable and accessible format?

Yes

Are the protocols appropriate and is the work technically sound?

Yes

Reviewer Expertise:

Internet of Things and Wireless sensor network

I confirm that I have read this submission and believe that I have an appropriate level of expertise to confirm that it is of an acceptable scientific standard, however I have significant reservations, as outlined above.

# Response

**Published:** 2023-11-24

**Authors:** Valters Abolins

**Affiliations:** Institute of Electronics and Computer Science, Latvia

Dear Prasanth A, thank you for the provided review and suggestions, here is our answers to them: 1. We have re-read and corrected the grammar in the paper as you suggested, leading to quite a large amount of edits improving the overall grammar and fluency of the paper. 2. We have improved the description about the Figure 1 in the main text stating more clearly what is depicted there.
